# Multi-Model Estimation Based Moving Object Detection for Aerial Video

**DOI:** 10.3390/s150408214

**Published:** 2015-04-08

**Authors:** Yanning Zhang, Xiaomin Tong, Tao Yang, Wenguang Ma

**Affiliations:** School of Computer Science, Northwestern Polytechnical University, Xi’an 710129, China; E-Mails: zhangyanning_npu@163.com (Y.Z.); yangtaonwpu@163.com (T.Y.); chogorima@gmail.com (W.M.)

**Keywords:** aerial video, object detection, multi-model estimation, Graph Cuts

## Abstract

With the wide development of UAV (Unmanned Aerial Vehicle) technology, moving target detection for aerial video has become a popular research topic in the computer field. Most of the existing methods are under the registration-detection framework and can only deal with simple background scenes. They tend to go wrong in the complex multi background scenarios, such as viaducts, buildings and trees. In this paper, we break through the single background constraint and perceive the complex scene accurately by automatic estimation of multiple background models. First, we segment the scene into several color blocks and estimate the dense optical flow. Then, we calculate an affine transformation model for each block with large area and merge the consistent models. Finally, we calculate subordinate degree to multi-background models pixel to pixel for all small area blocks. Moving objects are segmented by means of energy optimization method solved via Graph Cuts. The extensive experimental results on public aerial videos show that, due to multi background models estimation, analyzing each pixel’s subordinate relationship to multi models by energy minimization, our method can effectively remove buildings, trees and other false alarms and detect moving objects correctly.

## 1. Introduction

Moving target detection for aerial video is one of the core technologies of UAV (Unmanned Aerial Vehicle) surveillance systems. This technology can be widely applied in military domains such as battlefield reconnaissance and surveillance, positioning and adjustment, damage assessment, electronic warfare, *etc.* Also, it can support civil purposes such as border patrol, nuclear radiation detection, aerial photography, aerial prospecting, disaster monitoring, traffic patrol, security surveillance, *etc.* Due to its wide application, low cost, high cost effectiveness, no risk of casualties, strong survival ability, good maneuvering performance and convenience, moving object detection algorithm for UAV aerial video has become a hot research topic in the computer field. Moving object detection from a UAV is an important research topic crossing image processing and vehicle control. The purpose of this research is to automatically obtain the target position and motion information based on aerial video. This study can not only make UAV’s eyes more clear, but also guarantee the advanced processing and applications, such as behavior analysis and importance analysis.

We are faced with core difficulties in moving object detection for aerial video, such as motion mutation caused by UAV fast motion, low resolution noisy images, small target, low contrast, complex background, scale changes and occlusion, *etc.* With UAV development, researchers have proposed many algorithms to solve the above problems. However, most of these methods are under the registration-detection framework, which assumes that scenario only has a single background and will identify all the regions generating parallax error as targets. As a result, tracking failure usually happens in complex scenarios with multiple backgrounds, trees, buildings, *etc.* Therefore, the state of the art solutions in moving object detection cannot satisfy application need and it is developing new technology for complex scenes is necessary.

Automatic estimation of multiple background models for complex scenarios can provide a solution for perceiving the scene accurately. This paper first focuses on automatic estimation of multiple background models for complex scenarios. Then the pixels’ motion information and subordinate degrees to multi-background models are analyzed by optical flow. The subordinate degree between a pixel and a background model refers to the degree a pixel and its correspondence fit the background model. Usually, the projection error can be used to measure the subordinate degree. The larger the projection error, the lower the subordinate degree is. Based on the neighborhood information and the subordinate degree, we segment the moving objects via energy minimization [1,2]. Since we estimate multiple background models and perceive complex scenes correctly, our method can detect moving objects accurately under viaducts and other complex backgrounds. Meanwhile, our algorithm can effectively remove buildings, trees and other false alarms and improve the locating precision. In addition, the adoption of energy minimization, which makes use of both the analysis of neighborhood continuity and subordinate degree, can significantly improve segmentation precision.

The rest of this paper is organized as follows. Section 2 summarizes and analyzes the related work in recent years. Section 3 proposes the moving object detection algorithm based on multi-model estimation for aerial video of complex scenarios. The experimental results are reported in Section 4, which demonstrate the accuracy and effectiveness of our approach. Finally, the conclusions are drawn in Section 5.

## 2. Related Work

Moving object detection for aerial video [3] has widely developed in the past few decades. The existing moving object detection algorithms for aerial video mainly include two categories [4,5]: one is the bottom-up method and the other one is the top-down method. The bottom-up method is also named as Data-driven method, which does not rely on prior knowledge and extracts the moving information directly from the image sequences. Top-down method, also named the model-driven algorithm, which relies on the constructed model or prior knowledge, performs the matching computing and solves the posterior probability in image sequences. In matching computing, the moving objects will be detected if the similarity distance is close enough. When computing the posterior probability, the state vector corresponding to the maximum posterior probability will be denoted as the current status of the moving objects.

Using bottom-up method to realize moving object detection for aerial video mainly includes three steps [6,7,8,9,10]. The first step is image matching [11,12,13], which performs the adjacent frames registration for image sequences. The second step is object detection. Frame difference or background difference is often used to detect change blobs and obtain moving objects after registration. The third step is object classification. There are two tasks in this step. One is to extract the detected moving objects. The other one is to recognize these objects.

**Figure 1:**
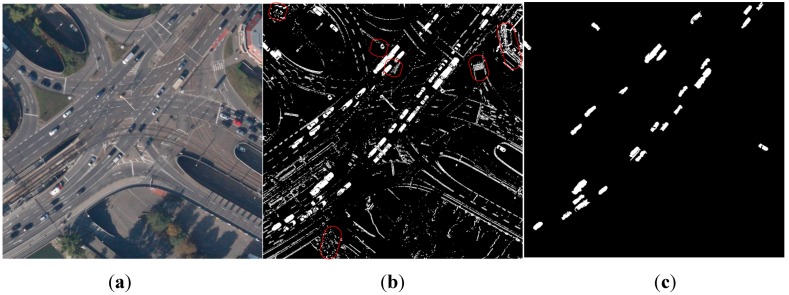
The comparison of moving detection results by different methods. (**a**) Original image (**b**) Moving detection with false alarms in red circles when using one affine model to describe the scene (**c**) Moving detection by our method.

The existing bottom-up algorithms for moving object detection include the classic COCOA system [14]. The procedure of this system contains image stabilization, frame difference and block tracking. However, this algorithm often fails in scenario scaling due to the Harris corner-based image stabilization. Cohen *et al.* [15,16] proposed a moving object detection and tracking system. First they aligned the images by estimating the affine transformation model iteratively. Then, the normalized optical flow field was applied for motion detection and the graph representation was constructed to resolve and maintain the dynamic template of moving objects. This system runs fast but it cannot solve the complex scaling scenarios. Ibrahim *et al.* [17] proposed the MODAT framework. Instead of Harris corner, they adopted SIFT (Scale-invariant feature transform) [18] features to fulfill the image matching. However, all of the above methods can only deal with simple background scenes and assume that only the moving objects can cause the parallax error. They tend to go wrong in complex multiple background scenarios, such as viaducts, buildings and trees. Chad *et al.* [19] proposed a moving object detection method for aerial video with low frame rate. They constructed an accurate background model to solve the object detection and the shadow problems. However, the application of this method is restricted because we need to know the camera calibration parameters in advance and start tracking objects manually. Shen *et al.* [20] proposed a moving object detection method for aerial video basing on spatiotemporal saliency. However, this method still cannot overcome the parallax error problem and the false alarm rate is high in complex scenarios. As shown in Figure 1b, false alarms (labeled by the red circles) occurred at buildings and trees when using one affine model to describe the scene. The real objects may be missed due to the inaccurate model estimation.

The top-down method transforms the moving object detection problem to Bayesian prediction. With the known prior probability of the object state, the problem can be solved by estimating the maximum *posteriori* probability continuously after obtaining the new measurement. In other words, Bayesian theory considers the vision-tracking problem as a “best guess” or “deduction” process, and usually adopts the state space approach to achieve vision tracking. The Classical Kalman filter [21] can only handle linear, Gaussian and unimodal situation. However, *posteriori* estimation is often non-linear, non-Gaussian and multimodal in practice. Therefore, EKF (Extended Kalman Filter) [22] is proposed to handle such cases. A particle filter [23] can also solve such non-linear problems.

The top-down method utilizes the *priori* knowledge to construct a model for the detection problem. Then, the model’s correction is verified with the practical image sequences. Since it has a solid theoretical foundation of mathematics and many mathematical tools that can be adopted, the top-down approaches are always the mainstream methods for vision detection. These approaches transform object detection problems to deduction and prediction problems. The assumption is that when the prior knowledge of deduction is correct, the deduction results will be correct. Otherwise, the results may be wrong. Thus, acquiring correct prior knowledge is very important. Existing approaches mostly initialize the objects manually to ensure the correctness of later subsequent detection and location, which is unrealistic in the practical applications. Therefore, in order to detect moving objects automatically for aerial video, reliable detection results from the bottom-up approach should be used as the deduction’s *priori* knowledge to achieve a correct prediction.

In this paper, we propose a moving object detection algorithm based on multi-model estimation for aerial video. First, we segment the scene into several color blocks and estimate the dense optical flow. Then, we calculate an affine transformation model for each large area block and merge the consistent models. Finally, Graph Cuts [1,2] is utilized to classify the foreground pixels into different objects. Our method can not only handle the moving object detection in the complex multiple background scenarios with viaducts, but can also remove buildings, trees and other false alarms effectively. As a result, the segmentation and detection precision will be improved.

## 3. Multi-Model Estimation Based Moving Object Detection

In order to overcome the influence of the complex multiple background scenarios, this paper proposes a moving object detection algorithm for aerial video basing on multi-model estimation. 

Firstly, the scene is segmented into several color blocks. Secondly, the affine transformation model between each background region in the current frame and the corresponding region in the previous frame is estimated basing on the dense optical flow. Thirdly, subordinate degree is calculated between each pixel and multiple background models to judge whether the pixel belongs to a moving object or not. Finally, moving objects are segmented by energy optimization method solved via Graph Cuts.

### 3.1. Algorithm Flow

**Figure 2:**
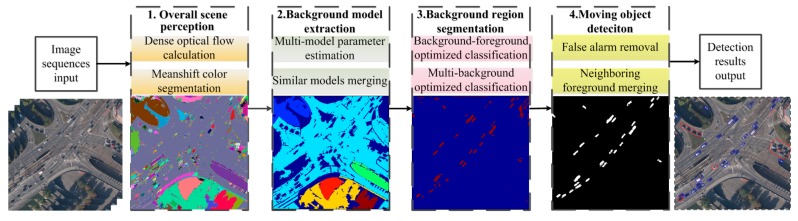
The flowchart of moving object detection based on multi-model estimation.

The flowchart of the proposed framework is shown in Figure 2. Our approach mainly includes four steps: the overall perception of the scene, background model extraction, background region segmentation and moving object detection. First, the overall perception of the scene segments the scene into several color blocks and estimates the dense optical flow. Here, the Mean shift pyramid segmentation method from OPENCV (Open Source Computer Vision Library) is adopted for color blocks segmentation and the Gunnar Farneback algorithm [24] is used for calculating dense optical flow. Second, to confirm the multiple background models included in the scenario, background model extraction calculates the affine transformation models for multiple color blocks and merges the consistent models. Third, the background region segmentation will be transformed to the background and foreground binary classification, multiple background regions and multiple labels classification problem. This problem can be solved by the energy optimization method, which can achieve smooth and continuous global optimal solution. Fourth, after obtaining the foreground regions, we merge the blocks and remove false objects based on the moving consistency and the region proximity. Afterwards, the moving object detection is finished and the accurate detected results are obtained. The background model extraction, background region segmentation and moving object detection are introduced in Section 3.2, Section 3.3 and Section 3.4, respectively. The details are as follows.

### 3.2. Multi-Model Estimation

Estimating accurately the background model parameters of complex scenarios can ensure the correct scene perception, accurate object segmentation and robust object tracking. The current multi-model estimation methods, like JLinkage [25], do not need any prior segmentation information and can classify samples into multiple categories automatically, where each category corresponds to one model. However, this method only adapts to small samples and is unable to solve the big samples like multi-model estimation under complex scenarios. In the aerial video, the background blocks with consistent color often belong to the same background and the background area is much larger than that of objects. Therefore, this paper first segments the scenarios into color blocks and selects the blocks with the large area as the candidate background blocks. Afterwards, an affine transformation model is estimated for each background block.

Let us denote *I_t_* and *I_t+_*_1_ as the adjacent two frames. Then, the dense optical flow can be computed by the Gunnar Farneback algorithm [24]. We define *OFX_t_* and *OFY_t_* as transverse and longitudinal optical flow, respectively. The corresponding relationships are as follows:

(1) $$I_{t}(x,y)\text{\textasciitilde{}}I_{t+1}(x\prime ,y\prime )$$

(2) $$x\prime =x+OFX_{t}(x,y),y\prime =y+OFY_{t}(x,y)$$

where $I_{t}(x,y)$ represents the pixel value in (x,y) of image *I_t_*. $I_{t+1}(x\prime ,y\prime )$ is defined as the pixel value in (x′,y′) of image *I_t+_*_1_. (x,y) and (x′,y′) form an optical flow pair.

Next, we segment *I_t_* by using Mean shift algorithm, which segments the scene into multiple color blocks based on their color consistency. Then, the blocks whose area is larger than threshold $T_{a\min}$ are selected as background blocks $B_{t}=\{b_{1},b_{2},...,b_{BNum}\}$, where BNum represents the number of the color background blocks obtained by segmenting. The color blocks’ area set is defined as $A_{t}=\{a_{b1},a_{b2},...,a_{bBNum}\}$ and $a_{bi}$ is the number of the pixels included in the *i*th background color block. Afterwards, each point in color background blocks and its optical flow point in the next frame obtained by optical flow method [24] form a point pair. Basing on the point pairs in each background block, the affine transformation model between the background block in the current frame and the corresponding region in the next frame is estimated via RANSAC (RANdom SAmple Consensus) method [12].

(3) $$M_{t}=\{m_{1},m_{2},...,m_{BNum}\}$$

(4) $$m_{i}=\left[\begin{array}{ccc} a_{2} & a_{1} & a_{0}\\ b_{2} & b_{1} & b_{0} \end{array}\right]$$

The affine transformation model set $M_{t}$ is composite of each background block’s affine transformation model. The affine transformation model of the *i*th background block is denoted as $m_{i}$, including translation, rotation, scaling, cropping and other atomic transformations. $a_{0}$ and $b_{0}$ represent shift amount between the background block in the current frame and the region in the next frame along the horizontal and vertical direction, respectively. The rest parameters represent composite of scaling, rotation and shearing. The current background blocks segmentation is based on color consistence, so single background may be segmented into several backgrounds due to color inconsistence. For the convenience of later scene analysis, we need to merge multiple background models according to the consistency between different background models. Thus, we define the projection error of the pair of points as follows:

(5) $$Error_{i}={\left\|{\left(x\prime ,y\prime \right)}^{T}-m_{i}\cdot {\left(x,y,1\right)}^{T}\right\|}_{2}$$

where (x′,y′) denotes the optical flow point of pixel (x,y) in the consecutive frame. The projection error is the difference in pixels, between two points located in consecutive images that are related by the optical flow. If $Error_{i}<T_{e}$, the point pair often belongs to the inliers of the model $m_{i}$, otherwise the pair of points is an outlier for the *i*th background block. Then we calculate the connective matrix $R_{BNum\times BNum}$ between the background blocks and the affine models as follows:

(6) $$R=\left[\begin{array}{cccc} r_{11} & r_{12} & \cdots & r_{1BNum}\\ \vdots & \vdots & \ddots & \vdots \\ r_{BNum1} & r_{BNum2} & \cdots & r_{BNumBNum} \end{array}\right]$$

(7) $$r_{ij}=\frac{a_{ij}}{a_{j}}$$

where $r_{ij}$ represents the accordance degree of the *j*th background block $b_{j}$ to the *i*th model $m_{i}$. $aI_{ij}$ is the number of inliers belonging to model $m_{i}$ in the *j*th background block $b_{j}$. $r_{ij}=\frac{aI_{ij}}{a_{bj}}$, denotes the *j*th background block’s rate of the inliers to model $m_{i}$. If $r_{ij}>T_{r}$ and $r_{ji}>T_{r}$, the *i*th background block $b_{i}$ and the *j*th background block $b_{j}$ are from the same background plane and can be combined to one background model. Thus, we update $B_{t}$ and $M_{t}$, st. $b_{i}=b_{i}\cup b_{j}$, and meanwhile delete $b_{j}$ and $m_{j}$.

### 3.3. Background Segmentation Based on Graph Cuts

We define the set of points that do not belong to the large background region as Ω. Then points of Ω can be judged as background region points or not based on the existing multiple background models. This paper proposes an energy minimization based algorithm for optimized classification. First, we define the scenario points belonging to l=BNum+1 categories, where *BNum* is the number of background models. We need to define and solve a label function f:Ω→L where L={0,1,2,...,BNum} are all the possible category labels for all the points in Ω. Label i>0 corresponds to the background pixels, which are located in the *i*th background region. Label 0 corresponds to no background models, but corresponds to the foreground pixels. Given a pixel *p*, if f(p)>0, it belongs to background region. Otherwise if f(p)=0, this pixel belongs to foreground region. Energy function is as below:

(8) $$E(f)=E_{d}(f)+E_{s}(f)$$

where data term $E_{d}$ represents the sum of classification cost of the points in Ω classified into different labels. The smooth term is a regularizer that encourages the neighboring pixels to share the same label. Therefore, the classification problem is transformed to minimizing E(f) and finding corresponding solution. However, minimizing E(f) directly is very difficult because the above classification problem is the coupling of foreground and background, and background and background classification. This paper decomposes the above problem into two optimized solution modules f={fs,fc}: (1) optimizing fs for background segmentation; (2) optimizing fc for classifying different background categories. In the first module, in order to segment the background regions, we transform this optimized classification problem to solve the binary energy minimization. If a pixel belongs to background, its label is 0, otherwise 1. The energy function includes a one variable data term and pairwise smoothing terms, where data term represents the cost of labeling the pixels to the background. The smoothing term corresponds to the continuous smoothness prior of the background region. The Graph Cuts [1,2] is adopted for optimizing and solving energy minimization problem. In the second module, the problem of classifying background points into different background models is transformed to a multi-labeling energy minimization problem, which can also be solved via Graph Cuts [1,2]. The data term of energy function represents the cost of tagging the points with the background labels. The smoothing term represents the background regions’ continuity constraint.

#### 3.3.1. Optimal Segmentation of Background Region

Following the above analysis, we need to seek a labeling function fs:Ω→Ls, fs:Ω→Ls. The background energy function is defined as follows:

(9) $$E(fs)=E_{d}(fs)+E_{s}(fs)$$

- Data term

If a point belongs to the background region, it should be an inlier of one background model and its projection error corresponding to background model should be small, otherwise this point belongs to the foreground region and is the outlier to all the background models. Therefore, we choose the projection error to define the data term $E_{d}(fs)$:

(10) $$E_{d}(fs)=\sum_{p\in \text{Ω}}\left|fs(p)-(1-Inl(p))\right|$$

(11) $$Inl(p)=\{\begin{array}{cc} 1 & \sum_{i=1}^{BNum}IsI_{i}(p)>0\\ 0 & otherwise \end{array}$$

(12) $$IsI_{i}(p)=\{\begin{array}{cc} 1 & Error_{i}(p)<T_{e}\\ 0 & otherwise \end{array}$$

where $IsI_{i}(p)$ represents pixel *p*’s inlier property projected in the model $m_{i}$. If the property is 1, this pixel belongs to the inliers, otherwise the outliers. Inl(p) represents pixel *p*’s background property. If property is 1, this pixel belongs to the background region, otherwise the foreground region. The penalty is given when pixel *p* is classified to the foreground point and Inl(p)=1. The classified cost is not 0 and fs(p)−(1−Inl(p))=1. Similarly, the classification penalty will also be given when the pixel *p* is classified to the background point and Inl(p)=0.

- Smooth term

Smooth term $E_{s}(fs)$ is a regularizer that encourages the overall labeling smoothly [1,2]. The prior is that two neighboring pixels have a higher probability to be classified as background points together or foreground points together. Here, we adopt the standard four-connected neighborhood system and penalize the fact if two neighboring pixels’ labels are different.

(13) $$E_{s}(fs)=\sum_{p\in \text{Ω},q\in N_{p}}S_{p,q}(fs(p),fs(q))$$

(14) $$S_{p,q}(fs(p),fs(q))=\min(\tau_{s},\beta (p,q)\cdot \left|fs(p)-fs(q)\right|)$$

(15) $$\beta (p,q)=h(\left|\overset{BNum}{\underset{i=1}{\min}}(Error_{i}(p))-\overset{BNum}{\underset{i=1}{\min}}(Error_{i}(q))\right|)$$

where $\tau_{s}$ and β(p,q) represent the maximum value and the weight of the smooth term, respectively. $N_{p}$ is the four neighborhoods of pixel *p*. h(⋅) is the weight function. The weight function is a decreasing function because a big penalty should be given when the neighboring pixels are classified to different categories if their minimum projection errors are similar. When the minimum projection errors of two pixels are more similar, the weight is bigger and the smooth constraint is stronger. The inverse function h(⋅) is selected to achieve smooth constraint. If two neighboring pixels *p* and *q* share the same label, then fs(p)=fs(q), $S_{p,q}(fs(p),fs(q))=0$. That is to say, the smooth cost is 0. Otherwise, if the neighboring pixels *p* and *q* are labeled with different tags, then the smooth cost $S_{p,q}(fs(p),fs(q))>0$. Just as defined in Equation (15), the closer the minimum projection errors of the two neighboring pixels, the bigger the smooth cost of labeling them with different tags.

Based on the design of above data term and smooth term, Graph Cuts is adopted to solve the minimization problem of E(fs). Afterwards, background segmentation result is obtained.

#### 3.3.2. Optimal Classification of Different Backgrounds

Denoting ${\text{Ω}}_{b}=\{p:|fs(p)=0\}$ as the set of points classified as the background pixels in Ω. We need to seek the labeling function $fc:{\text{Ω}}_{b}\to Lc$,Lc={1,2,...,BNum}. Similarly, we adopt the energy minimization framework for solving fc. The energy minimization of the background classification is defined as follows:

(16) $$E(fc)=E_{d}(fc)+E_{s}(fc)$$

- Data term

Data term should reflect the subordinate degree between background pixel and multi-background models, and achieve minimum value if the pixel belongs to someone model. Projection error can satisfy above requests. Therefore, we define the cost function by using projection error as follows:

(17) $$E_{d}(fc)=\sum_{p\in {\text{Ω}}_{b}}Errop_{fc(p)}(p)$$

- Smooth term

Smooth term $E_{s}(fs)$ is a regularizer that encourages the overall labeling is smooth [1,2]. Similar with Section 3.3.1, we adopt the standard four-connected neighborhood system and penalize if the labels of two neighboring pixels are different.

(18) $$E_{s}(fc)=\sum_{p\in {\text{Ω}}_{b},q\in N_{p}}S_{p,q}(fc(p),fc(q))$$

(19) $$S_{p,q}(fc(p),fc(q))=\{\begin{array}{cc} \min(\tau_{s},\beta (p,q)) & fc(p)\neq fc(q)\\ 0 & otherwise \end{array}$$

where $\tau_{s}$ and β(p,q)have the similar definitions to Section 3.3.1. We also adopt Graph Cuts to minimize E(fc) and obtain the segmentation of different backgrounds. The points of ${\text{Ω}}_{b}$ are classified to the corresponding background blocks according to classified results of the label function fc.

(20) $$b_{i}=b_{i}\cup \{\forall p\in {\text{Ω}}_{b},p:|fc(p)=i\}$$

### 3.4. Moving Object Detection

**Figure 3:**
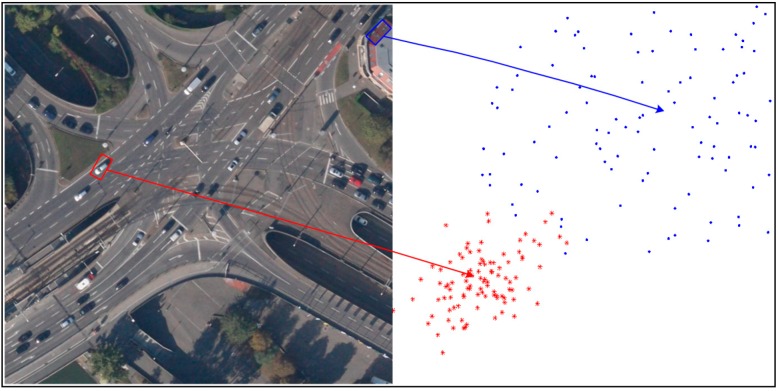
The distribution of motion vectors in blocks.

The pixels classified as foreground pixels may come from true moving object, and may also belong to false alarms of parallax error caused by buildings and others. How to distinguish these two category points is the key of segmenting moving object accurately. As we know, when a moving object is compensated by the background model, the parallax error only causes by the object itself, which represents the absolute motion vector of the object. Then the object motion between two neighboring frames is approximately the linear motion. As a result, the motion vectors of the inliers belonging to one object are similar. As shown in Figure 3, the motion vectors of true object in the red bounding box are similar. In contrast, the buildings do not belong to any background and all the existing background models cannot compensate the parallax error caused by the platform motion. Therefore, no matter if it is compensated by any one of the background models, parallax error distributes without dissimilarity, as the false alarm in the blue box of Figure 3.

According to the above analysis, we will first calculate the motion vectors of foreground blocks compensated by the background model, and determine the moving objects by analyzing similarity of the motion vectors. The final foreground color blocks $Ob_{t}=\{ob_{1},ob_{2},...,ob_{ObNum}\}$ can be obtained by integrating the foreground-background segmentation in Section 3.3 and the color segmentation in Section 3.2. Here, ObNum is the color block number of the current foreground regions. We count the labeling set of the background models surrounding each color block and denote the labeling set as $MOb_{t}=\{mob_{1},mob_{2},...,mob_{ObNum}\}$, where $mob_{j}=\{i:|\exists p\in ob_{j},q\in b_{i},p\in N_{q}\}$. The number of labels in $mob_{j}$ is $MNum_{j}$. The motion vector of pixel p(x,y) after compensated by model *m_i_* is defined as follows:

(21) $$\vec{v}={\left(x\prime ,y\prime \right)}^{T}-m_{i}\cdot {\left(x,y,1\right)}^{T}$$

For each color block $ob_{j}$, we calculate the motion vector set compensated by each model, and count the mean value and variance of Gaussian distribution of the motion vector set. If the variance is small enough ($T_{\sigma }$ is the variance threshold), this block is a moving block, otherwise a false object. The details of the procedure are in Algorithm 1.

**Algorithm 1** False Object Removal Algorithm Input: background model *T_σ_*, motion block set *Ob_t_* and model label set *MOb_t_*. Output: motion blocks with false alarms removed.
FOR j=1:ObNum DOFOR$\;i=1:MNum_{j}$ DOComputing the motion vectors compensated by corresponding models for the *i*th label, which belongs to labels of pixel $mob_{j}$ in $ob_{j}$Counting the mean value $\;\vec{\mu_{i,j}}$ and variance $\;\vec{\sigma_{i,j}^{2}}$ of these motion vectors.ENDIf $\underset{i}{\min}|\vec{\sigma_{i,j}^{2}}|>T_{\sigma }$ or the area of $ob_{j}$ is larger than threshold $T_{a\max}$, $ob_{j}$ is the false alarm block and should be removed, otherwise $ob_{j}$ is background block and we define the label of minimum variance model as $j_{m}$. Then the background model label that $ob_{j}$ belongs to is also set to $j_{m}$. The moving speed of block $\vec{v_{j}}=\vec{\mu_{j_{m},j}}$ and the corresponding variance $\vec{\sigma_{j}^{2}}=\vec{\sigma_{j_{m},j}^{2}}$.END

Therefore, we can obtain the foreground blocks by removing false alarm and updating $Ob_{t}$ and ObNum. However, these blocks are segmented using the color consistence. Since the object color may be inconsistent, sometimes an object will be segmented into several blocks. To overcome this drawback, we need to merge these foreground blocks. We calculate the adjacent matrix $Nb_{ObNum\times ObNum}=\left\{nb_{jk}\right\}$ between moving blocks, where $nb_{jk}=1$ represents that the *j*th and *k*th foreground block are neighborhood, *i.e.*, $\exists p\in ob_{j},q\in ob_{k},q\in N_{p}$. $nb_{jk}=0$ indicates that two blocks are not neighborhood. Next we calculate the area sum matrix $A_{ObNum\times ObNum}=\left\{a_{jk}\right\}$ of moving blocks. If the sum of *j*th foreground block area and the *k*th foreground area $a_{j}+a_{k}<T_{a\max}$, then $a_{jk}=1$, otherwise 0. We compute the speed similarity matrix $V_{ObNum\times ObNum}=\left\{v_{jk}\right\}$. If the background model label of *j*th and the *k*th foreground block are same as well as ${\left|\vec{v_{j}}-\vec{v_{k}}\right|}^{2}<\left|\vec{\sigma_{j}}\right|\cdot \left|\vec{\sigma_{k}}\right|$, then $v_{jk}=1$, otherwise 0.

If ∃j,k, *st*. $n_{jk}\cdot a_{jk}\cdot v_{jk}=1$, then we consider that the *j*th block and the *k*th foreground block belong to the same object and merge them to one. The new moving speed of the union foreground block is $\frac{a_{j}\cdot \vec{v_{j}}+a_{k}\cdot \vec{v_{k}}}{a_{j}+a_{k}}$ and the corresponding variance is recalculated. Nb, *A* and *V* also need to be recalculated.

Afterwards, merging is repeated until no foreground blocks can be merged. The final merged results are the moving object detection results.

## 4. Experimental Results and Analysis

In order to evaluate the proposed multi-model estimation based moving object detection algorithm for aerial video, we perform the comparison experiments on the public DAPAR VIVID (Defense Advanced Research Projects Agency, Video Verification of Identity program) and KIT AIS (Karlsruher Institut für Technologie Aerial Image Sequences) Data Set databases. In DAPAR VIVID database [26], the EgTest01 dataset contains many moving cars but the background is relatively simple. In KIT AIS Data Set [27], shooting frame rate is 1FPS and it includes viaducts, overpasses, buildings, trees and other complex scenarios, which is very challenging for the moving object detection algorithms of aerial video. The configuration of the computer used in our experiments is CPU Intel(R) Core(TM) 2 Duo 2.66 GHz, RAM 2.0 G. It takes about 4 s to process each frame for 724 × 708 image sequences. The most time-consuming step is the Mean shift segmentation, which takes about 3.5 s per frame. Dense optical flow calculation takes about 0.25 s and Graph cuts takes about 0.25 s. We have not done any acceleration. For practical application, parallel computing and other fast calculation method can be used to accelerate the segmentation and detection procedure. Our approach involves several parameters, including mainly the background threshold $T_{a\min}$, the object area threshold $T_{a\max}$, the projection error threshold $T_{e}$, the variance threshold $T_{\sigma }$ and the smooth threshold $\tau_{s}$. The color blocks with area larger than $T_{a\min}$ are considered to be background blocks. The smaller $T_{a\min}$ is set, the more background models we get, and the more complicated the multi-model estimation step is. The bigger $T_{a\min}$ is set, the more likely we miss some background models. In our experiments, we set $T_{a\min}=6400$ to get a balance between the complexity and model number. $T_{a\max}$ is the max threshold for the object area. If it is set too small, then true object will be considered as small background blocks. Otherwise, the objects close to each other would be considered as one with large value for $T_{a\max}$. In our experiments, we set $T_{a\max}=800$ to detect vehicles on the road. $T_{e}$ is the projection error threshold. If a pixel’s projection error for a given affine model is bigger than $T_{e}$, then it is considered to be an outlier for the model. Otherwise, if its projection error is smaller than $T_{e}$, it is an inlier for this model. The smaller $T_{e}$ can bring more outliners and meanwhile cause more false alarms. The bigger $T_{e}$ sometimes makes the algorithm miss true moving pixels. For the balance of false alarms and missing, we set $T_{e}=\sqrt{3}$ in our experiment. The variance threshold $T_{\sigma }$ determines which foreground blocks are true object blocks and which blocks are false alarms. The smaller the value of $T_{\sigma }$, the fewer false alarms we detect and meanwhile the more likely we miss the true moving object. The larger $T_{\sigma }$ would cause more false alarms. We set $T_{\sigma }=4$ in our experiment for the best performance. The smooth threshold $\tau_{s}$ defines the max smooth cost of labeling two neighboring pixels with different tags. The larger $\tau_{s}$ brings a smoother labeling map and object missing is more likely to occur. The small $\tau_{s}$ decreases the smoothing effect. We set $\tau_{s}=4$ in our experiments.

The detection method in [14] is the most representative method in which Harris features are abstracted for registration and frame difference is used to detect moving objects. Shen *et al.* [20] proposed a moving object detection method for aerial video based on spatiotemporal saliency. This method can accurately handle moving target detection under simple scenarios. However, it has not adopted multiple background analysis for the scenarios, and detection missing and false alarms will happen frequently in complex scenarios. As there are no published codes for the approach in [14,20] on the web, we implement these two approaches for comparison.

**Figure 4:**
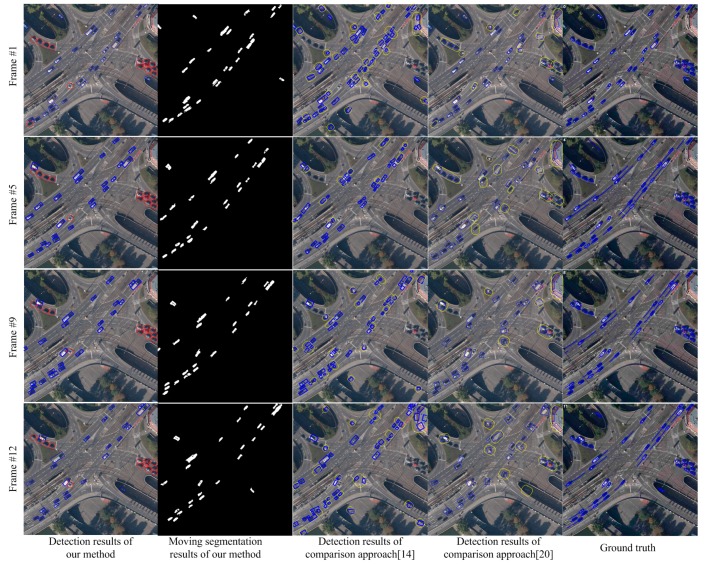
Detection comparisons in complex overpass scenarios.

We compare our algorithm with the method in [14] and Shen [20] on the StuttgartCrossroad01 dataset of KIT AIS Data Set. The results are shown in Figure 4. This dataset contains overpasses and multiple background complex scenarios as well as complex elements, such as trees and shadow, which will influence the detection results. All the factors will bring substantial challenge to the detection algorithms. In Figure 4, the images from top to bottom show the detected results of the 1st, 5th, 9th and 12th frames. The images from left to right are separately, the detection results of this paper, the segmentation results of this paper, the detection results by [14], the detection results of Shen [20], and the ground truth. In the first column of Figure 4, the objects in blue boxes are the detection results of this paper. The objects in red boxes are stationary targets. The detection results show our approach can segment and detect moving objects accurately in the complex background situation of overpasses. Since both of the approaches, in [14,20], cannot perceive multiple background of the scenario and cannot obtain accurately background information, the situations such as inaccurate moving segmentation and false alarms will happen. We can see these situations in the third and fourth column of Figure 4. The blue bounding boxes show the detected objects. The yellow boxes show the false detection and missing targets. Although, the method in [20] performs better than the method in [14], false alarms and inaccurate detections occur frequently in both of these two methods. The ground truth published on the web marks all the vehicles in the scene, including both moving objects and stationary vehicles.

**Figure 5:**
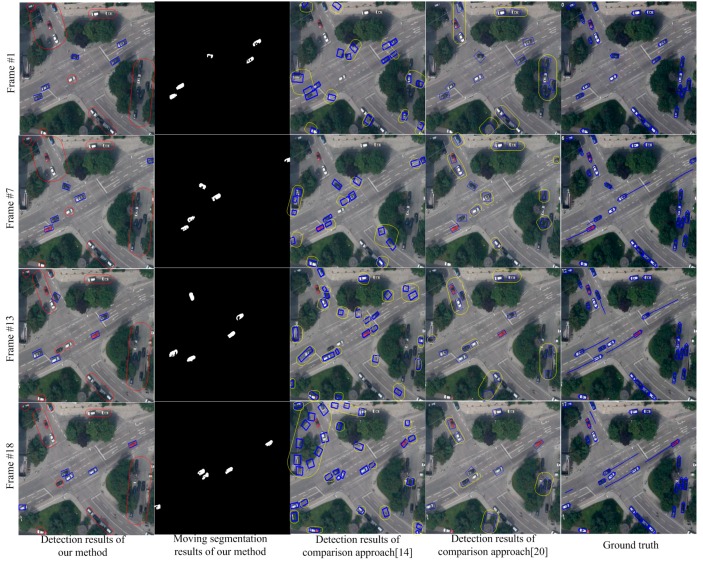
Detection comparisons in scenarios with many trees.

**Figure 6:**
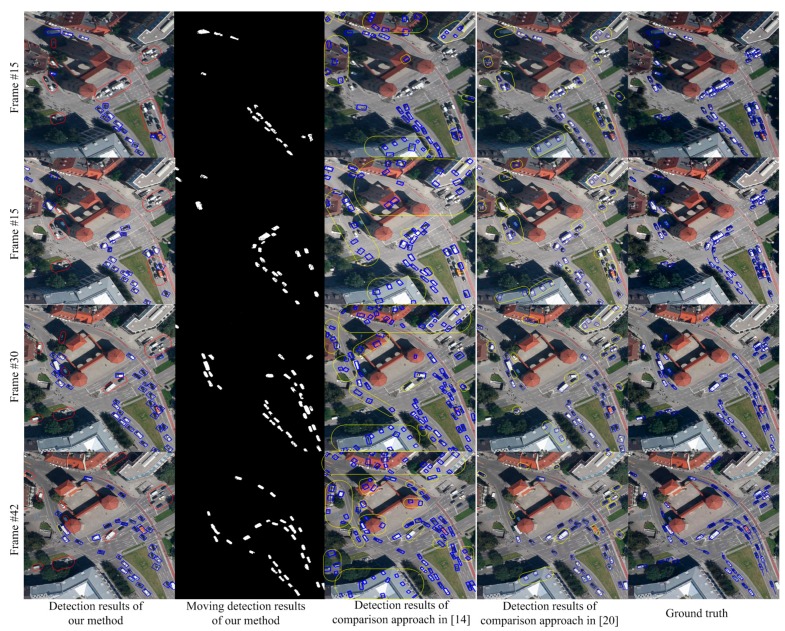
Detection comparisons in scenario with many buildings.

Figure 5 shows the comparison results on the MunichCrossroad01 dataset. The characteristic of this dataset is that the false objects of parallax error caused by the trees and other elements occupy a large proportion of the image area. In Figure 5, the images from top to bottom show the detection results of the 1st, 7th, 13th and 18th frames. The results in the first column show that our approach can handle the moving object detection in scenarios with many trees and overcome the parallax error caused by trees. In contrast, the traditional detection methods [14,20] based on registration will be influenced by trees and cannot estimate the scene model accurately. Therefore, as shown in the third and fourth columns of Figure 5, the detection rate of traditional method is low and the false alarm is high. Figure 6 shows the detection results on Munich Crossroad02 dataset. This dataset includes many buildings. The transitional methods [14,20] cannot accurately estimate the background parameters and obtain the correct detection and segmentation results in this situation. As shown in the third and fourth columns of Figure 6, many false alarms and missing detections occur. In contrast, the results in the first and second columns show the detection and segmentation results of our paper. The results demonstrate our approach can perceive scenarios and detect moving objects correctly due to multiple background model estimation.

As shown in Figure 4, Figure 5 and Figure 6, this paper performs much better than traditional detection algorithm basing on registration. Our approach can analyze the multiple background models in scenarios and detect the moving objects accurately. However, since we adopt mean shift color segmentation and pyramid dense optical flow to perceive the multiple background models, the algorithm’s efficiency still needs to be improved and more efficient multiple background model estimation algorithms are required. Additionally, this paper focuses on vehicle-sized objects and cannot detect the point objects like humans. We also do not add any special treatment for shadow, so the moving objects after segmenting may contain shadow, which is also the future work.

**Figure 7:**
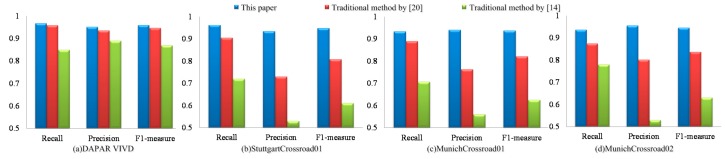
The statistical result of our method and the traditional methods by [14,20].

In order to quantitatively analyze the detection accuracy of this paper, we define recall *R*, accuracy *P* and comprehensive evaluation indicators F1 as follows:

(22) R=DObNum/ObNum

(23) P=DObNum/DNum

(24) F1=2PR/(P+R)

where ObNum, DObNum and DNum are the object number of the ground truth, correct detection and detected object number, respectively. If a detected object’s overlap rate with a true object is above 0.5, then it is considered as a correct detection. Otherwise, it is a false alarm. In practical applications, higher *R* and *P* are desired, but these two indicators are contradictory in some cases. F1 integrates the results of *R* and *P*. Higher F1 indicates that the experimental method is more effective. Figure 7 shows the comparison results of our paper, the traditional method by [20] and the traditional method by [14]. The results from left to right are the statistical results of DAPAR VIVID EgTest01, StuttgartCrossroad01, MunichCrossroad01 and MunichCrossroad02 of KIT AIS Data Set. As shown in Figure 7a, these three algorithms can both achieve high detection rate under simple background and their detection precisions are similar. However, F1 of our algorithm under complex background is higher than the methods in [14,20], *i.e.*, on StuttgartCrossroad01 dataset, F1 of our result is 0.949, which is higher than 0.808 of Shen [20] and 0.611 of the method in [14]. In MunichCrossroad01 dataset, our approach’s F1 is 0.937, which is higher than 0.821 of Shen [20] and 0.625 of the method in [14]. These results show the significant superiority of our algorithm, as shown in Figure 7b–d.

## 5. Conclusions

This paper is mainly for the moving object detection problem under complex scenarios for aerial videos. We propose a novel moving object detection algorithm based on multi-model estimation and optimized classification. First, we calculate the dense optical flow of the scene and do color segmentation basing on mean shift to capture the perception of the whole scene. Secondly, we calculate affine transformation models as the multiple background models for each color block with a large area. Through multiple background model cross-validation and merger, accurate multi-model parameters of scene can be obtained. Thirdly, in order to obtain the multiple background segmentation results of the scene, the background points are segmented into multiple background models by using energy optimization method solved via Graph Cuts. Finally, we calculate subordinate degree from foreground regions to multi-background models, remove the false alarm and segment moving object accurately.

Since we break through the single background constraint and adopt multiple background models, our algorithm can handle the moving object detection under complex multiple background scenarios. Moreover, our algorithm can segment the background and foreground regions accurately due to the adoption of Graph Cuts, optical flow information and continuous smooth constraints. The experimental results on many aerial videos indicate that our algorithm can correctly perceive multiple background information of the scene and detect moving object accurately in the complex scenes with multiple backgrounds, buildings and other objects that produce parallax.
